# Genetic Polymorphisms Associated with Vincristine Pharmacokinetics and Vincristine-Induced Peripheral Neuropathy in Pediatric Oncology Patients

**DOI:** 10.3390/cancers14143510

**Published:** 2022-07-19

**Authors:** Mirjam E. van de Velde, Aniek Uittenboogaard, Wenjian Yang, Erik Bonten, Cheng Cheng, Deqing Pei, Marleen H. van den Berg, Inge M. van der Sluis, Cor van den Bos, Floor C. H. Abbink, Marry M. van den Heuvel-Eibrink, Heidi Segers, Christophe Chantrain, Jutte van der Werff ten Bosch, Leen Willems, William E. Evans, Gertjan J. L. Kaspers

**Affiliations:** 1 Emma Children’s Hospital, Amsterdam UMC, University of Amsterdam, 1081 HV Amsterdam, The Netherlands; a.uittenboogaard@amsterdamumc.nl (A.U.); mh.vandenberg@amsterdamumc.nl (M.H.v.d.B.); g.j.l.kaspers@prinsesmaximacentrum.nl (G.J.L.K.); 2 Department of Pharmaceutical Sciences, St. Jude Children’s Research Hospital, Memphis, TN 38105, USA; wenjian.yang@stjude.org (W.Y.); erik.bonten@stjude.org (E.B.); william.evans@stjude.org (W.E.E.); 3 Princess Máxima Center for Pediatric Oncology, 3584 CS Utrecht, The Netherlands; i.m.vandersluis@prinsesmaximacentrum.nl (I.M.v.d.S.); c.vandenbos-5@prinsesmaximacentrum.nl (C.v.d.B.); m.m.vandenheuvel-eibrink@prinsesmaximacentrum.nl (M.M.v.d.H.-E.); 4 Department of Biostatistics, St. Jude Children’s Research Hospital, Memphis, TN 38105, USA; cheng.cheng@stjude.org (C.C.); deqing.pei@stjude.org (D.P.); 5 Emma Children’s Hospital, Amsterdam UMC, Amsterdam Medical Center, Pediatric Oncology, 1105 Amsterdam, The Netherlands; f.abbink@amsterdamumc.nl; 6 Department of Pediatric Hemato-Oncology, University Hospitals Leuven and Catholic University Leuven, 3000 Leuven, Belgium; Heidi.segers@uzleuven.be; 7 Department of Pediatrics, Clinique du MontLégia, CHC, 4000 Liège, Belgium; christophe.chantrain@chc.be; 8 Department of Pediatric Onco-Hematology, Universitair Ziekenhuis Brussel, 1090 Brussels, Belgium; jutte.vanderwerfftenbosch@uzbrussel.be; 9 Department of Paediatric Haematology-Oncology and Stem Cell Transplantation, Ghent University Hospital, 9000 Ghent, Belgium; leen.willems@uzgent.be

**Keywords:** neurotoxicity, children, cancer, vincristine, toxicity, DNA, single-nucleotide polymorphism, area under the curve, maximum concentration, whole-exome sequencing

## Abstract

**Simple Summary:**

Vincristine is a type of chemotherapy that is often used in the treatment of children with cancer. The main side effect of vincristine is nerve damage. Patients experience symptoms such as tingling, pain or muscle weakness. Some children are more sensitive to vincristine than others, which may depend on variations in genes and in the breakdown of vincristine by the body. In this study, we investigated the effect of variations in genes on nerve damage due to vincristine and breakdown of vincristine by the body. We found that nine variations in seven genes were associated with nerve damage due to vincristine, whereas three variations in three genes were associated with the breakdown of vincristine by the body. It is important that future studies try to replicate these findings. Our findings help us towards the goal of tailoring vincristine treatment to each child, with optimal therapeutic effect while limiting nerve damage.

**Abstract:**

Vincristine (VCR) is an important component of curative chemotherapy for many childhood cancers. Its main side effect is VCR-induced peripheral neuropathy (VIPN), a dose limiting toxicity. Some children are more susceptible to VIPN, which is at least partially dependent on genetic factors and pharmacokinetics (PK). In this study, we identify and replicate genetic variants associated with VCR PK and VIPN. Patient samples from a randomized clinical trial studying the effect of administration duration of VCR on VIPN in 90 patients were used. PK sampling was conducted on between one and five occasions at multiple time points. A linear two-compartment model with first-order elimination was used, and targeted next-generation DNA sequencing was performed. Genotype–trait associations were analyzed using mixed-effect models or logistic regression analysis for repeated measures, or Poisson regression analysis in which the highest VIPN score per patient was included. Nine single-nucleotide polymorphisms (SNPs) in seven genes (NDRG1, GARS, FIG4, FGD4, SEPTIN9, CEP72, and ETAA1) were associated with VIPN. Furthermore, three SNPs in three genes (MTNR1B, RAB7A and SNU13) were associated with PK of VCR. In conclusion, PK of VCR and VIPN are influenced by SNPs; upfront identification of those that lead to an altered susceptibility to VIPN or VCR exposure could help individualize VCR treatment.

## 1. Introduction

Vincristine (VCR) is one of the oldest and most widely used chemotherapeutic agents in pediatric oncology. It works by inhibiting mitosis by restriction of microtubule formation in the mitotic spindle [1,2]. It is metabolized in the liver by the cytochrome P450 (CYP) family of enzymes into the active metabolite M1, mainly by CYP3A4 and CYP3A5. The main dose limiting type of toxicity is VCR-induced peripheral neuropathy (VIPN). It is a mixed motor and sensory neuropathy, affecting the distal part of the longer nerves (i.e., feet and hands) and progressing more proximally when the condition worsens. It causes symptoms of paresthesia, pain, numbness or muscle weakness, among others. It also affects the autonomic nervous system where it can lead to, for example, constipation. VIPN is associated with a lower quality of life, both in patients undergoing treatment and in survivors of childhood cancer [3,4]. 

There are several factors that influence the development of VIPN in children receiving VCR. In terms of administration related factors, it was shown that a single dose exceeding 1.5–2 mg/m^2^ with a maximum of 2 mg resulted in intolerable toxicity in general [5]. Furthermore, the development of VIPN is influenced by administration duration of VCR [6,7] and the individual pharmacokinetics (PK) of VCR [8,9]. In terms of patient related factors, older age appears to be associated with a higher risk of VIPN, although findings have been conflicting [2]. The relation between sex and VIPN remains unclear [2]. Moreover, it was shown that ancestry is associated with VIPN development, with studies showing that Caucasian children are more often affected than African(-American) children [10,11]. It was thus hypothesized that genomic factors are associated with VIPN [11]. Initially, this research focused on genomic differences between the CYP3A4 and CYP3A5 enzymes, since there was an ancestry dependent difference in the distribution of protein expression (i.e., African-American children express more CYP3A5 and Caucasian children express more CYP3A4). CYP3A5 was associated with faster metabolization and lower exposure to VCR [10,12,13,14,15,16], which could possibly explain the lower rate of VIPN in African-American children. However, these results were conflicting and could not fully explain the individual differences between VIPN [11]. In 2015, a genome-wide association study (GWAS) was published that identified a single-nucleotide polymorphism (SNP) in the gene coding for the Centrosomal Protein 72 (CEP72) that was associated with VIPN development [5]. Other studies have replicated this association since then [17,18]. To summarize all pharmacogenomic parameters associated with VIPN, we recently performed a systematic review and meta-analysis in which we found that SNPs in transporter-, metabolism-, cytoskeleton-, and hereditary neuropathy-associated genes were associated with VIPN [11].

Although the relation between genomic factors and VIPN has frequently been studied, all associated factors cannot fully explain the individual differences in the development of VIPN. The exposure of VCR after an administration is also determined by pharmacokinetics (PK), which is in turn affected by genetic factors. However, the direct effect of genetic variants on PK of VCR has not yet been studied. Understanding the association between genetic factors and PK is important, as it could lead to an individualized dosing regimen of VCR [9,11,19]. For example, children with a genetic variant associated with a higher exposure to VCR could benefit from dose reductions, whereas children who have a lower exposure might not benefit from the generally applied dose capping. By taking both PK and genetic associations into account, the individual dose of VCR could be optimized, while chances of developing VIPN are minimized [19]. Therefore, the goal of our study was to investigate the association between genetic factors and PK of VCR and to replicate or identify genetic factors associated with VIPN in pediatric oncology patients.

## 2. Materials and Methods

### 2.1. Patients 

Data of patients were collected as part of a randomized controlled clinical trial (RCT) which aimed to study the association between administration duration of VCR and VIPN in pediatric oncology patients. The design and results of this randomization are described elsewhere [20]. Briefly, participants of this RCT received all planned VCR administrations of their treatment protocol either through intravenous push injections (in 1–5 min) or through one-hour infusions. Children with the following diseases and treatment protocols were eligible for participation: acute lymphoblastic leukemia (ALL) (DCOG ALL-11 protocol 11 protocol [21], EORTC-58081-CLG guideline [22] or EsPhALL protocol [23]), Hodgkin lymphoma (EuroNet-PHL-C1 protocol [24] or C2 protocol [25]), rhabdomyosarcoma (EpSSG RMS 2005 protocol [26]), nephroblastoma (SIOP Wilms 2001 protocol [27]), medulloblastoma (ACNS0331 [28] or ACNS0332 [29] protocol), and low-grade glioma (SIOP LGG 2004 protocol [30]). Blood samples for PK analysis were collected between September 2014 and April 2018 in either one of four Dutch or three Belgian participating centers. The study was approved by the Institutional Review Board of the VUmc. Of all participants, written informed consent was obtained from parents and/or children (in case the child was ≥12 years). Participants of the RCT could choose and declare on the informed consent form whether they also wanted to participate in the PK part of the study.

### 2.2. Genomic Analysis

During treatment germline DNA of each participant was collected as specified in the study protocol. Whole blood samples were collected in PAXgene DNA collection tubes (Qiagen, Mississauga, ON, Canada). DNA was isolated and purified using the PAXgene Blood DNA Kit (Qiagen, Mississauga, ON, Canada) according to the manufacturer’s instruction. DNA was analyzed using a targeted approach studying 48 candidate genes. These genes were selected based on a previously studied relation with VIPN, a relation with Charcot-Marie Tooth (CMT) (an inherited form of peripheral neuropathy which can worsen after VCR administration), possible relation with VCR PK, or possible association with VIPN based on the function of the expressed gene. These 48 genes are displayed in Supplementary materials S1. For each candidate gene, we included all exons and putative regulatory regions of 10,000 base pairs flanking the gene for targeted sequencing (Supplementary materials S1). Therefore, in this discovery and replication study, both SNPs that have previously been described in relation to VIPN and new SNPs were studied. The putative regulatory regions were selected based on ENCODE data version 3. Specifically, we extracted enhancer, transcriptional factor binding, and DNAse I hypersensitive regions for each cell line in ENCODE. Those regions presenting all three features in multiple cell lines were selected. In addition, we also included regions 500 bp surrounding cis-acting expression quantitative trait loci (eQTLs) from the GTEx Portal (version 6, *p*-value < 10^−7^). Roche NimbleGene SeqCap EZ probes were designed to capture these regions (Roche, Roche NimbleGen, Madison, WI, USA). Illumina HiSeq 2000 was used for paired-end sequencing with 100 bp read length. Sequencing reads in FASTQ format were mapped and aligned using the Burrows-Wheeler Aligner, and genetic variations were jointly called following GATK best practice version 3.8. Only those genotypes passing GATK quality control and exhibiting call rates greater than 95% were included for further association analysis. Linkage disequilibrium (LD) pruning was performed using the ‘plink’ package (r^2^ ≥ 0.30) [31,32]. The estimated impact of missense variants on protein function was estimated in silico using the scaled Combined Annotation Dependent Depletion (CADD) score [33] and the Rare Exome Variant Ensemble Learner (REVEL) score [34]. For intronic SNPs, the predicted impact on splicing was estimated in silico using the deep-learning-based tool SpliceAI [35].

### 2.3. Pharmacokinetic Analysis

Methods and results of the PK analysis were previously published [9]. Briefly, blood samples were collected at 10, 20, 30, 40, 60, 75, 140 and, if the child was still in the hospital, 1440 min after start of VCR treatment. Depending on the length of the treatment protocol, samples were taken at 1–5 different occasions during the treatment period. Samples were analyzed using high-performance liquid chromatography (HPLC)/tandem mass spectrometry (HPLC/MS/MS). The assay quantifies VCR concentrations in plasma from 0.25 to 100 ng/mL. A linear two-compartment model with first-order elimination was used to describe the VCR concentration vs. time data. The model was fit to PK data from all individuals simultaneously using non-linear mixed-effect modeling (Monolix, version 5.1.0 with the stochastic approximation expectation-maximization (SAEM) method. For this study, the included individual post-hoc PK estimates (Empirical Bayesian Estimates (EBE)) were used to estimate the plasma VCR area under the concentration time curve (AUC) and maximum VCR concentration (C_max_).

### 2.4. Assessment of VIPN

VIPN was assessed prospectively at between one and five occasions during treatment using two different instruments. Of the Common Toxicity Criteria of Adverse Events (CTCAE) (version 4.03 [36]) the items peripheral sensory neuropathy (grade 0–5), peripheral motor neuropathy (grade 0–5), constipation (grade 0–5) and neuralgia (grade 0–3) were used to calculate a VIPN sum score. A CTCAE item score of two or higher was defined as VIPN. Furthermore, the Dutch translated version of the pediatric-modified Total Neuropathy Score (ped-mTNS) [37] was used. This validated instrument, which includes both a questionnaire part (sensory, functional and autonomic symptoms) and a physical examination, has been developed to assess VIPN in children aged 5 years or older. As such, in the current study this instrument was not used in children below 5 years of age. A total ped-mTNS score of ≥5 was defined as VIPN [37]. 

### 2.5. Statistical Analysis

**Figure 1:**
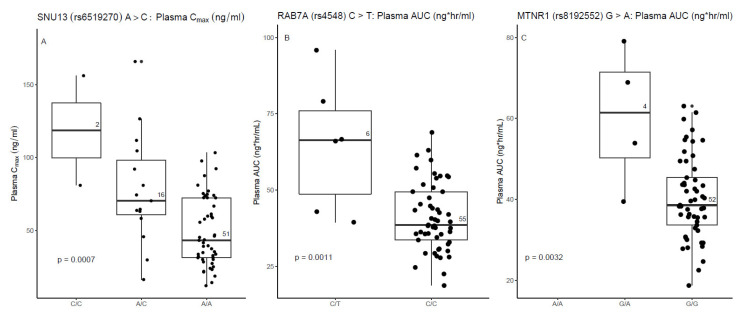
(**A**) Association between a SNP in SNU13 and vincristine (VCR) pharmacokinetics (PK). (**B**) Association between a SNP in RAB7A and VCR PK. (**C**) Association between a SNP in MTNR1 and VCR PK. PK samples were collected in 35 patients on maximum 70 occasions; every occasion per patient is shown. The number in the boxplot indicates the number of observations per genotype. The *p*-value was derived from mixed-effect linear regression for repeated measures, where the genotype was considered to be a categorical variable. SNU13: Small Nuclear Ribonucleoprotein 13, RAB7A: RAS-related protein 7A, MTNR1B: Melatonin Receptor 1B.

**Figure 2:**
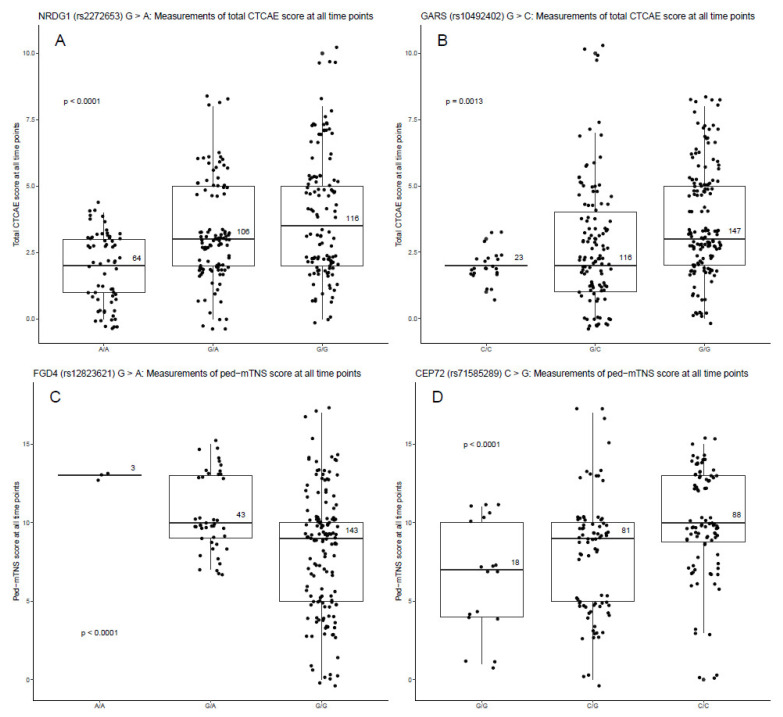
(**A**) Association between a SNP in NRDG1 and total Common Toxicity Criteria of Adverse Events (CTCAE) score. (**B**) Association between a SNP in GARS and total CTCAE score. (**C**) Association between a SNP in FGD4 and pediatric-modified Total Neuropathy Score (ped-mTNS). (**D**) Association between a SNP in CEP72 and ped-mTNS score. VIPN measurements were performed 1–5 times in 85 patients. Every VIPN measurement per patient across the time points is shown. The number in the boxplot indicates the number of observations per genotype. The *p*-value was derived from Poisson regression analysis for repeated measures, where the genotype was considered to be a categorical variable. NRDG1: N-Myc Downstream Regulated 1, GARS: Glycyl tRNA Synthetase, FGD4: FYVE, RhoGEF and PH Domain Containing 4, CEP72: Centrosomal Protein 72.

**Figure 3:**
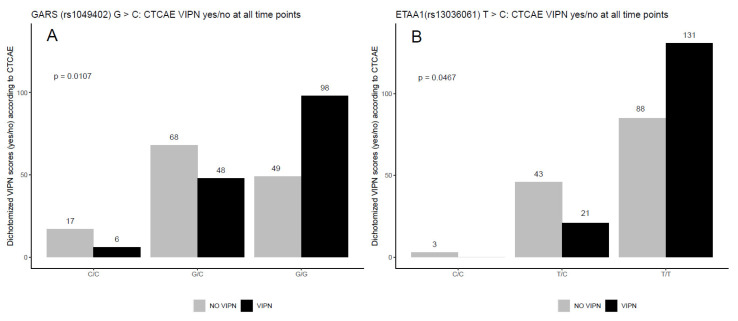
(**A**) Association between a SNP in GARS and dichotomized (yes/no) VIPN scores according to the CTCAE score. (**B**) Association between a SNP in ETAA1 and dichotomized (yes/no) VIPN scores according to the CTCAE score. VIPN measurements were performed 1–5 times in 85 patients. Every VIPN measurement per patient across the time points is shown. The number in the boxplot indicates the number of observations per genotype. A cut-off value of a CTCAE score of ≥2 was considered to be VIPN. The *p*-value was derived from mixed-effect logistic regression for repeated measures. For GARS, genotype was considered to be a categorical variable, whereas for ETAA1, genotype was considered to be an ordinal variable. ETAA1: Ewing’s tumor-associated antigen 1.

VCR plasma PK parameters C_max_ and AUC were generated longitudinally at multiple occasions. Their associations with SNP genotypes were analyzed by mixed-effect linear models for repeated (longitudinal) measurements with genotype as the main effect of interest and a random effect with compound-symmetry intra-subject correlation structure. For a clear visualization, gross associations between the PK parameters and genotypes were displayed in box plots by pooling all measurements together ignoring time (Figure 1). Furthermore, VIPN was measured in two ways: according to the CTCAE, defined as the sum of the CTCAE grades of all VIPN-related toxicities, and the ped-mTNS. Mixed-effect Poisson regression models were fitted for the highest total CTCAE and total ped-mTNS scores per patient across the time points with SNP genotypes as the main effect of interest, and the baseline total CTCAE or ped-mTNS score as a covariate. For clear visualization, the total CTCAE and ped-mTNS scores across the different time points were displayed by boxplots according to genotypes (Figure 2 and Figure S1). Second, the dichotomized VIPN scores (VIPN yes/no, defined CTCAE ≥ grade 2 on VIPN-related toxicities) were modeled by mixed-effect logistic regression, including the baseline CTCAE or ped-mTNS score as a covariate, and with a random effect to account for intra-subject correlation. Again, for clear visualization, the number of VIPN assessments (yes/no) per patient were shown in boxplots according to genotype (Figure 3). To correct for multiple hypothesis testing, a false discovery rate (FDR) correction was applied to determine a significance threshold (*p* < 0.004, FDR = 23%) [38]. A multivariable analysis was performed, in which the additional variables disease, cumulative VCR dosage, and ancestry were included.

## 3. Results

### 3.1. Study Population

In total, 90 patients participated in the RCT, of whom 85 had sufficient DNA material available for analysis and thus were included in the DNA cohort in which the association between genetic variations and VIPN was described. For the CTCAE, 286 measurements were available in 85 patients with a median of four measurements per patient (interquartile range (IQR): 1–4). For the ped-mTNS, measurements were available in 59 patients with a median of three measurements per patient (IQR: 3–4). Furthermore, 35 out of 90 patients participated in the PK part of the trial (*n* = 70 occasions, 425 samples). These patients were included in the PK cohort in which the association between genetic variations and PK parameters was described. Patient characteristics are displayed in Table 1. 

### 3.2. Genomic Analysis

The genotype data on a total of 767 SNPs were available and out of these, 263 SNPs had a minor allele frequency (MAF) of over 5%. After LD pruning (r^2^ ≥ 0.30), the number of SNPs was reduced to 98. In the total PK group, the median AUC was 39.78 (ng·hr)/mL and median C_max_ was 57.28 ng/mL. Furthermore, 44 out of 90 (49%) patients developed VIPN based on CTCAE and 55 out of 66 (patients ≥ 5 years of age; 83%) developed VIPN based on ped-mTNS. In total, 12 SNPs of ten genes were nominally significantly associated with any of our outcomes which all except two SNPs passed stringent correction for multiple testing (*p* < 0.004, FDR = 23%). 

Three SNPs in three genes (Small Nuclear Ribonucleoprotein 13 (SNU13), RAS-related protein 7A (RAB7A), Melatonin Receptor 1B (MTNR1B)) were significantly associated with the PK of VCR (Figure 1). Patients with one or two minor alleles in these genes had higher plasma C_max_ or plasma AUC values (Table 2). The intronic SNP in SNU13 is an eQTL for SNU13 (Table S1). The estimated impact on protein function of the missense variant in MTNR1B was moderate (Table S1). None of the SNPs exhibited estimated splicing effects (Table S1).

**Figure 4:**
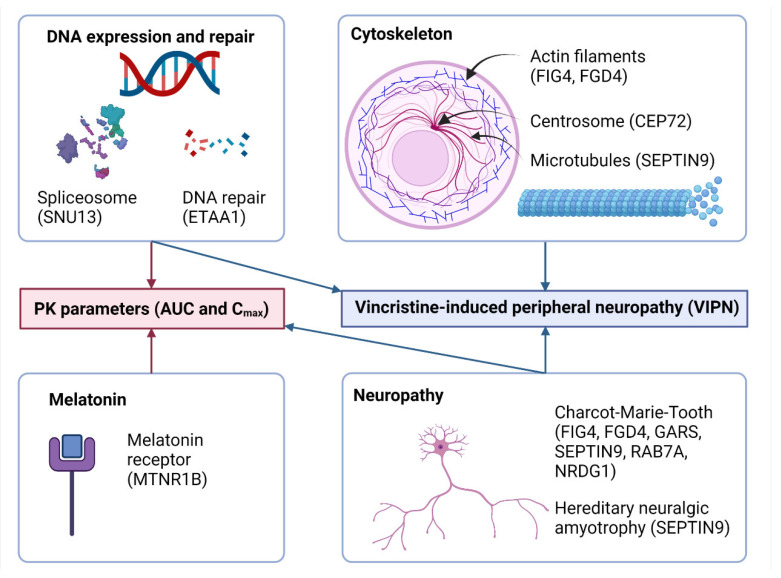
Schematic overview of the significant associations between genetic variations and VCR PK and VIPN. Figure created with Biorender.com.

Nine SNPs in seven genes were associated with total VIPN (Figure 2 and Figure S1) and dichotomized VIPN scores (Figure 3), of which six and three SNPs were associated with lower and higher VIPN scores, respectively (Table 2). A missense variant located in the transcription factor binding site for Glycyl tRNA Synthetase (GARS) was associated with both total and dichotomized CTCAE scores. Similarly, an intronic SNP that is an eQTL for ETAA1 was associated with both total ped-mTNS score and dichotomized CTCAE score (Table S1). Two untranslated regions (UTR) variants in FIG4 Phosphoinotiside 5-Phosphatase (FIG4) were associated with VIPN, one of which is a missense variant with estimated moderate deleteriousness for protein function and one is an eQTL for FIG4 (Table S1). For FYVE, RhoGEF and PH Domain Containing 4 (FGD4), two SNPs were significantly associated with VIPN, of which one is an eQTL for FGD4 (Table S1). Finally, three intronic SNPs in N-Myc Downstream Regulated 1 (NRDG1), Septin 9 (SEPTIN9), and Centrosomal Protein 72 (CEP72) were associated with VIPN. As with the SNPs associated with the PK values, none of the SNPs exhibited estimated splicing effects (Table S1).

The results for all associations were similar in the multivariable analysis (Figure S2). A systematic overview of the function of the genes with SNPs that had significant associations with PK or VIPN is presented in Figure 4. 

## 4. Discussion

In this study, we have shown that genetic variants are associated with important VCR PK variables in pediatric oncology patients. We also replicated previously identified genetic associations involving VIPN and we have identified new genetic variants associated with VIPN. Since VIPN is a dose limiting toxicity, leading to impaired health-related quality of life in children [3], it is of utmost importance to identify patients at risk of developing VIPN and where possible to modify treatment dose to mitigate toxicity. However, such dose modifications should not result in low VCR exposure that could compromise anticancer effects of VCR. It is therefore important to not only understand the association between VIPN and genetic variants, but also VCR PK and genetic variants. Our study is, to our knowledge, the first to assess the association between PK of VCR and genetic variants in children with cancer. 

Three SNPs in three genes were associated with the PK of VCR. First, we described a SNP that was an eQTL for SNU13, which is a highly conserved gene involved in pre-mRNA splicing as a component of the spliceosome [39]. Low expression of this gene is associated with increased sensitivity of primary leukemia cells to VCR, and concomitant use of SNU13 inhibitors and VCR both increases cytotoxicity of VCR and reduces VCR effects on neurons [40]. The reported SNP has not previously been described in relation to VIPN or PK of VCR. However, this specific eQTL variant may contribute to SNU13 expression variation and consequently affect the PK of VCR. Furthermore, a missense variant in MTNR1B was associated with the PK of VCR. MTNR1B is known for its role as coding for the melatonin receptor, but is also associated with other diseases such as type 2 diabetes [41,42]. Diouf et al. first described another SNP in MTNR1B in relation to VIPN [5]. Hypothetically, this association may be the result of PK differences between genotypes. The SNP found in this study has not been previously described in relation to VIPN or PK of VCR. Finally, a SNP in RAB7A was associated with the plasma AUC of VCR. RAB7A encodes for a protein that regulates vesicle traffic in the late endosomes and from the late endosomes to lysosomes. This SNP in RAB7A has been described in relation to CMT type 2 [43]. In addition, this SNP was a C to T transversion, a variation that may be the result of oxidative stress [44,45]. Oxidative stress is a significant cause of DNA damage, not only in cancer cells, but also in germline cells [44,45].

Nine SNPs in seven genes were associated with VIPN, of which four genes are related to the cytoskeleton (Figure 4). VCR exerts its cytotoxic effect via the inhibition of mitosis, by acting on the cytoskeleton of the cell, establishing a biologically plausible links between genes involved in the cytoskeleton and VIPN [1]. First, we found a SNP in CEP72, a gene encoding for a centrosomal protein that is required for adequate chromosome segregation. CEP72 in relation to VIPN was first described by Diouf et al. [5]. They found that a promotor variant in CEP72 was linked to VIPN in both children [5,17] and adults [46]. The role of this SNP was recently confirmed in a meta-analysis [18]. However, the impact of this SNP appears limited to the certain treatment phases, such as the continuation phase of ALL treatment, since the significant association was not replicated in the induction phase [47,48]. Of note, the SNP that we found in this study has not been previously described in relation to VIPN. It is not in LD with the SNP from Diouf et al. [5]. Nonetheless, we confirm the role of CEP72 in relation to VIPN in this study. Second, we found SNPs in FIG4 and FGD4, genes that are both involved in the regulation of the actin cytoskeleton and cell shape [49,50,51,52] and have been implicated in CMT type 4 [53,54]. For FIG4, one SNP that was associated with an increased risk of VIPN has been previously described in adults with multiple myeloma as a risk factor for VIPN [55]. Furthermore, one of the SNPs in FGD4 is a C to T transversion, which may be the result of oxidative stress, as described above [44,45]. Thirdly, we found a SNP in SEPTIN9, a gene that encodes for a protein involved in cytokinesis and cell cycle control via the microtubules [56,57]. The gene has been implicated with hereditary neuralgic amyotrophy [56,57]. The reported SNP in SEPTIN9 has not been described in relation to VIPN previously.

In addition to the SNPs described above, SNPs in two more genes have previously been described in relation to CMT or hereditary neuropathies (Figure 4). One SNP in NRDG1, a gene encoding for an intracellular protein that can be induced under a wide variety of stress and cell growth conditions, is associated with CMT type [58,59]. Furthermore, one SNP in GARS, a gene that has been identified as a causative gene responsible for the clinical features of distal hereditary motor neuropathies type 4, was seen in patients suffering from CMT or sensory neuropathy [53,60,61,62]. Interestingly, opposed to their findings, the minor allele was associated with less neuropathy in our study population.

One SNP in a gene involved in DNA repair was associated with VIPN, namely a SNP in ETAA1 (Figure 4). The biological function of ETAA1 is not well described, but it appears to function as a DNA replication stress response protein and thus is important for genome stability [63,64]. Diouf et al. described another SNP in ETAA1 in relation to VIPN [5], although this association was not replicated in the adult population [65].

To investigate the association between genetic variants and VIPN, the method of VIPN measurement is an important consideration. Previously, this was mostly done via retrospective CTCAE assessment [2]. However, it was shown that up to 40% of children with VIPN are not identified using this method [66]. New VIPN assessment tools have been developed, such as the ped-mTNS, which performs better than those historically used and are currently recommended for the measurement of VIPN in children [67,68]. In our study, we used specifically trained assessors for the prospective measurements of VIPN with two different tools, therefore identifying more children with VIPN symptoms than in previous studies. We thus used three different methods to identify children with VIPN, namely total CTCAE and ped-mTNS score and dichotomized CTCAE score. Of note, only two SNPs in GARS and ETAA1 had significant associations with two out of the three methods. As described above, the CTCAE and ped-mTNS measure symptoms of VIPN differently, which may explain this discrepancy. For further replication studies, it is important to take this into consideration. Moreover, it would be interesting to assess the effect of different dichotomization cut-off values, to study for example the effects of genetic variants on severe VIPN and any grade of VIPN compared to no grade of VIPN.

Our study has some limitations which should be considered. First, our sample size was small (*n* = 85) for assessing with adequate power the association between genetic polymorphisms and VIPN with adequate power and more so for genetic factors associated with PK (*n* = 35). Nonetheless, our results replicated previously reported genetic polymorphisms, which seems to endorse those previous reported associations. However, due to the exploratory nature of this study, the newly identified genetic factors should however be interpreted as preliminary and await external replication, since there was no independent replication cohort available in this study. In addition, although the stringent FDR threshold to correct for multiple hypothesis testing was met for most of our findings, the FDR rate was still 23%, consistent with the possibility of a substantial number of false positive findings. Nonetheless, many of our findings involve genes previously linked to neuropathy, providing increased confidence in their association with VIPN. 

The association between genetic factors and VIPN has frequently been studied. Many genetic factors appear to be associated with VIPN; however, as previously mentioned, results of the various studies are difficult to compare due to the diversity of the tools used to assess VIPN. This could be an explanation why many associations were only discovered in a single study and could not be confirmed in other studies. However, it may also reflect the multifactorial nature of VIPN [2]. Unfortunately, this means that the single SNPs associated with VIPN have a small effect size, because the development of VIPN is dependent on so many factors. Individualized VCR dosing strategies based on single SNPs aiming to reduce VIPN is therefore probably not feasible. Similarly, upfront screening of patients who will receive VCR based on single SNPs will not identify those at highest risk of developing (severe) VIPN. It is thus of importance to relate relevant SNPs to each other, to establish a genetic risk score. To identify those relevant SNPs, future studies would ideally perform a whole genome strategy in a sufficient sample size, corrected for relevant confounders such as cumulative VCR dosage, and with adequate prospective assessment of VIPN to study the association between genomic factors and VIPN. Furthermore, PK sampling should be part of this study to identify whether an identified SNP in a particular gene affects VIPN directly, or also affects VCR PK, since dose adaptation of VCR to prevent VIPN development should not result in VCR exposure below therapeutic efficacy. So far, no such study has been performed.

## 5. Conclusions

In conclusion, we identified and replicated several genetic associations between VCR PK and VIPN in children with cancer. The recognition that the occurrence of VIPN and VCR PK are associated with genetic polymorphisms in plausible genes provides insights that may prove useful in optimizing VCR treatment of children with cancer.

## Figures and Tables

**Table 1:** Patient characteristics of the included patients.

	Patients (Total Cohort) (*n* = 90)	Patients (PK Cohort) (*n* = 35)	Patients (DNA Cohort) (*n* = 85)
Age in years (mean, SD)	9.17 (5.15)	10.06 (5.60)	8.95 (5.00)
Ancestry (*n*, %)			
European	73 (81.11)	30 (85.71)	69 (81.18)
Non-European	17 (18.89)	5 (14.29)	16 (18.82)
Sex (*n*, %)			
Female	40 (44.44)	19 (54.29)	37 (43.53)
Male	50 (55.56)	16 (45.71)	48 (56.47)
Diagnosis (*n*, %)			
ALL	58 (64.44)	26 (74.29)	54 (63.53)
Hodgkin	18 (20.0)	6 (17.14)	18 (21.18)
Medulloblastoma	2 (2.22)	1 (2.86)	2 (2.35)
LGG	2 (2.22)	1 (2.86)	2 (2.35)
Wilms tumor	8 (8.89)	1 (2.86)	7 (8.24)
RMS	2 (2.22)	0 (0)	2 (2.25)
Mean (SD) cumulative VCR dose per m^2^	7.41 (7.99)	13.25 (9.36)	13.91 (9.23)
Mean (SD) AUC ((ng·hr)/mL)	N.A.	41.78 (14.32)	N.A.
Mean (SD) VCR C_max_ (ng/mL)	N.A.	57.44 (31.82)	N.A.
Median (IQR) total CTCAE score	1.00 (0.00–2.00)	1.00 (0.00–3.00)	1.00 (0.00–2.00)
Median (IQR) total ped-mTNS score *	4.00 (1.00–8.00)	4.00 (1.00–8.25)	4.00 (1.00–8.50)
Patients with VIPN according to CTCAE (%)	40 (44.4)	16 (45.71)	40 (47.06)

* Total group was *n* = 66 (no. of patients aged ≥5 years), PK: pharmacokinetics, DNA: deoxyribonucleic acid, SD: standard deviation, ALL: acute lymphoblastic leukemia, LGG: low-grade glioma, RMS: rhabdomyosarcoma, VCR: vincristine, area under the concentration time curve, N.A.: not available, C_max_: maximum plasma concentration of VCR, IQR: interquartile range, CTCAE: Common Terminology Criteria for Adverse Events, ped-mTNS: pediatric-modified Total Neuropathy Score, VIPN: vincristine-induced peripheral neuropathy.

**Table 2:** Associations between single-nucleotide polymorphisms (SNPs), VCR PK and VIPN.

Outcomes per SNP									Effect Size	
									Variant Heterozygous vs. Wild-Type	Variant Homozygous vs. Wild-Type
PK Outcomes	SNP				Wild-Type *(n of Patients)*	Variant Heterozygous Genotype *(n of Patients)*	Variant Homozygous Genotype *(n of Patients)*	*p*-Value	Regression Coefficient (95% CI)	
	Gene	RS-Code	Mutation	Consequence						
AUC	*MTNR1B* *	rs8192552	G > A	Missense NMD transcript variant	G/G (28)	G/A (2)	A/A (0)	0.0032	20.42 (8.0–32.8)	N.A.
	*RAB7A* *	rs4548	C > T	Synonymous (intron)	C/C (28)	C/T (2)	T/T (0)	0.0011	23.54 (10.9–36.2)	N.A.
C_max_	*SNU13* *	rs6519270	A > C	Non-coding transcript variant (intron)	A/A (23)	A/C (8)	C/C (1)	0.0029	26.72 (7.4–46.0)	69.55 (23.0, 116.1)
**VIPN Outcomes**	**SNP**				**Wild-Type (*n of Patients)***	**Variant Heterozygous Genotype *(n of Patients)***	**Variant Homozygous Genotype *(n of Patients)***	***p*-Value**	**Ratio of Mean (95% CI)**	
	**Gene**	**RS-Code**	**Mutation**	**Consequence**						
Total CTCAE	*NDRG1* *	rs2272653	G > A	Splice region variant (intron)	G/G (34)	G/A (28)	A/A (19)	<0.0001	0.84 (0.73–0.96)	0.49 (0.40–0.60)
	*GARS* *	rs1049402	G > C	Missense transcription factor binding site variant	G/G (43)	G/C (32)	C/C (6)	0.0013	0.71 (0.62–0.82)	0.47 (0.34–0.64)
Total ped-mTNS	*FIG4* *	rs9885672	T > C	Missense UTR variant of the 5′ UTR	T/T (40)	T/C (19)	T/T (0)	<0.0001	1.44 (1.30–1.59)	N.A.
	*FIG4* *	rs10659	G > A	UTR variant of the 3′ UTR	G/G (53)	G/A (6)	G/G (0)	<0.0001	1.53 (1.34–1.75)	N.A.
	*FGD4* *	rs12823621	G > A	Splice region variant (intron)	G/G (45)	G/A (13)	A/A (1)	<0.0001	1.43 (1.27–1.60)	1.88 (1.36–2.59)
	*FGD4* *	rs73083501	C > T	NMD transcript variant (intron)	C/C (39)	C/T (18)	T/T (2)	<0.0001	0.86 (0.76–0.97)	0.46 (0.32–0.68)
	*SEPTIN9* *	rs11650934	C > G	UTR variant of the 5′ UTR	C/C (41)	C/G (17)	G/G (1)	<0.0001	0.62 (0.54–0.71)	0.81 (0.48–1.38)
	*CEP72* *	rs71585289	C > G	Upstream gene variant (intron)	C/C (27)	C/G (25)	G/G (6)	<0.0001	0.84 (0.75–0.93)	0.53 (0.43–0.66)
	*ETAA1* *	rs35777125	G > A	Non-coding transcript variant (intron)	G/G (43)	G/A (15)	A/A (1)	0.0007	0.9 1 (0.82–1.02)	0.36 (0.19–0.70)
**Dichotomized VIPN Outcomes**	**SNP**				**Genotype (*n of Patients*)**	**VIPN + (*n of Observations*)**	**VIPN − (*n of Observations*)**	***p*-Value**	**Odds Ratio (95% CI)**	
	**Gene**	**RS-Code**	**Mutation**	**Consequence**						
VIPN (yes/no) according to CTCAE	*GARS* *	rs1049402	G > C	Missense transcription factor binding site variant	G/G (43)	98	49	0.0107	0.52 (0.28–0.99)	0.18 (0.03–0.92)
					G/C (32)	48	68			
					C/C (6)	6	17			
	*ETAA1* **	rs35777125	G > A	Non-coding transcript variant (intron)	G/G (62)	131	88	0.0467	0.31 (0.16–0.57)	
					G/A (18)	21	43			
					A/A (1)	0	3			

SNP: single-nucleotide polymorphism, CI: confidence interval, NMD = nonsense-mediated decay, UTR = untranslated region. * *p*-value indicates statistical significance of overall genotype effect on the phenotype as described by the regression model. For the PK parameters mixed-effect linear models were fit, a random effect was included to account for intra-patient (repeated measure) correlation. For the total grade by CTCAE or ped-mTNS (an integer-valued phenotype), Poisson regression model was fit. For the dichotomized VIPN by CTCAE, mixed-effect logistic regression model was fit. Apart from one exception (see footnote below), genotype was regarded as a categorical variable. ** Given the small number of homozygotes for the variant allele, OR was expressed as carriers of the variant allele (G/A and A/A) compared with homozygous wild-type (G/G). 4. Discussion.

## Data Availability

Data are available upon reasonable request due to privacy/ethical constrictions.

## Supplementary Materials

The following supporting information can be downloaded at: https://www.mdpi.com/article/10.3390/cancers14143510/s1, Supplementary materials S1: Selection of the 48 candidate genes that were analyzed in relation to VCR PK or possible association with VIPN based on the function of the expressed gene and the SNPs included in the analysis; Figure S1: Genetic variants significantly associated with VIPN according to the CTCAE and ped-mTNS; Figure S2: The estimates of the coefficients with and without adjusting for covariates (disease, cumulative VCR dosage and ancestry); Table S1: CADD en REVEL scores for all missense SNPs for predicted deleteriousness for protein function, delta score from SpliceAI for estimated impact on splicing and description of SNPs that are located in eQTL and their respective tissue types.
