# Supplementary material for: Genetic Polymorphisms Associated with Vincristine Pharmacokinetics and Vincristine-Induced Peripheral Neuropathy in Pediatric Oncology Patients
Source: Cancers (Basel). 2022 Jul 19;14(14):3510. doi: 10.3390/cancers14143510 (PMC9321338; doi:10.3390/cancers14143510)
Supplement: Supplementary file 1 [file cancers-14-03510-s001.zip › PGx VINCA Supplementary materials_18072022_V6.pdf]

Supplementary materials 2

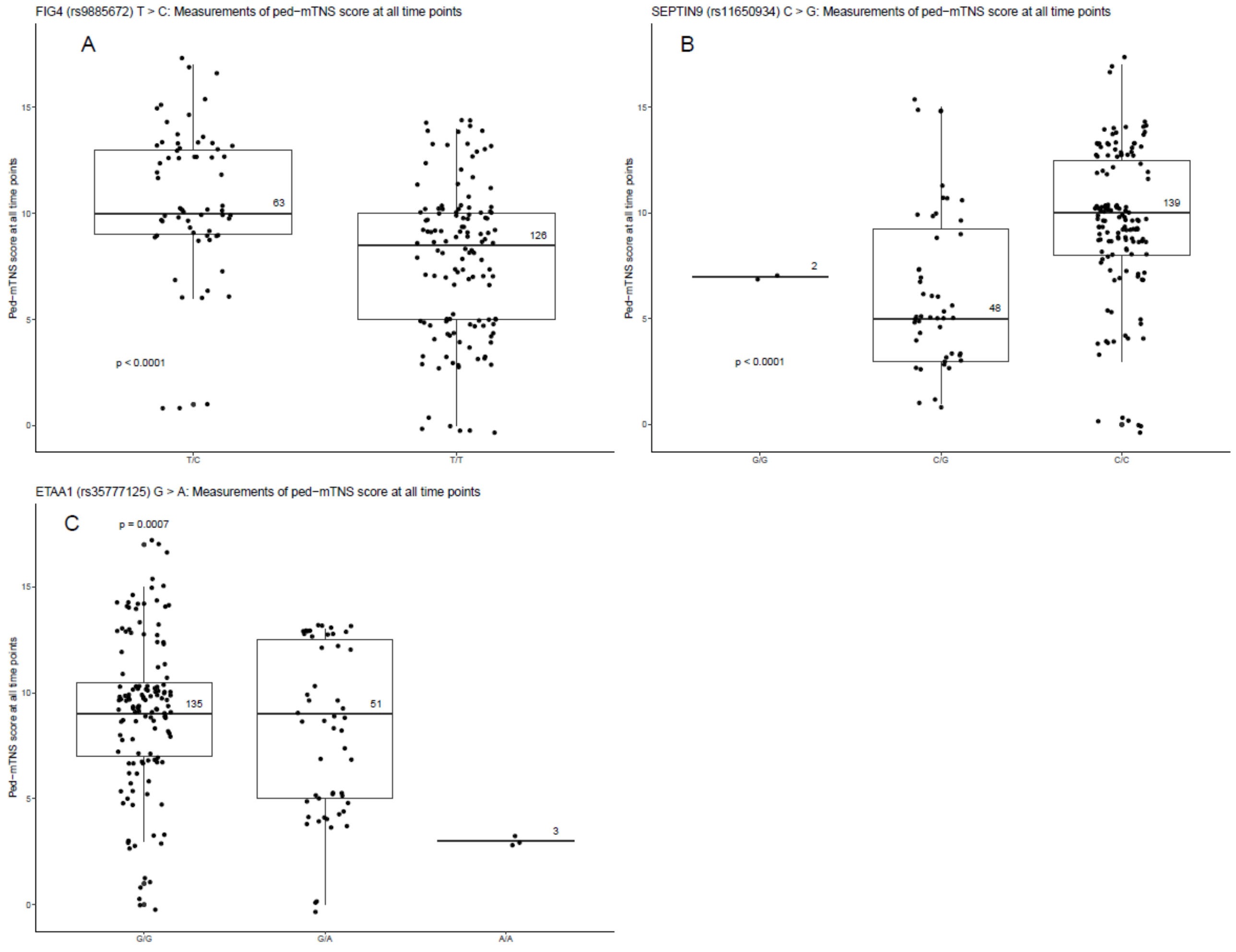

Figure S1. Genetic variants significantly associated with VIPN according to the CTCAE and ped-mTNS. VIPN measurements were performed 1-5 times in 85 patients. Every VIPN measurement per patient across the time points is shown. The number in the boxplot indicates the number of observations per genotype. The p-value was derived from Poisson regression analysis for repeated measures, where the genotype was considered as a categorical variable. FIG4: FIG4 Phosphoinositide 5-Phosphatase, SEPTIN9: Septin 9, CEP72: centrosomal protein 72, ETAA1: Ewing's tumor-associated antigen 1.

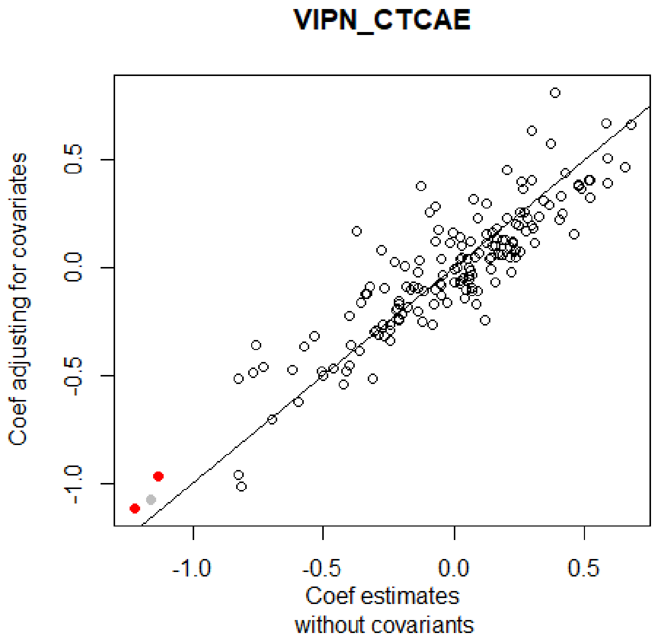

Figure S2. The estimates of the coefficients with and without adjusting for additional covariates (disease, cumulative VCR dosage and ancestry). Baseline CTCAE scores were included in both models. Red dots are SNP rs1049402 in GARS and SNP rs35777125 ETAA1, and the gray dots are two other ETAA1 SNPs pruned out.

Table S1. CADD and REVEL scores for all missense SNPs for predicted deleteriousness for protein function, delta score from SpliceAI for estimated impact on splicing and description of SNPs that are located in eQTL and their respective tissue types.

Gene	SNP	Missense or non-coding	CADD score	REVEL score	Splice delta score	eQTL for respective gene	Tissue type for eQTL
CEP72	rs71585289	Non-coding	N.A.	N.A.	0	No	N.A.
ETAA1	rs35777125	Missense	0.04	0.05	0	Yes	Tibial artery, adipose (visceral), skin
FGD4	rs12823621	Non-coding	N.A.	N.A.	0	No	N.A.
	rs73083501	Non-coding	N.A.	N.A.	0	Yes	Thyroid, testis
FIG4	rs9885672	Missense	3.35	0.20	0	No	N.A.
	rs10659	Non-coding	N.A.	N.A.	0	Yes	Heart
GARS	rs1049402	Missense	N.A.	N.A.	N.A.	No	N.A.
MTNR1B	rs8192552	Missense	3.42	0.28	0	No	N.A.
NDRG1	rs2272653	Non-coding	N.A.	N.A.	N.A.	No	N.A.
RAB7A	rs4548	Non-coding	N.A.	N.A.	0	No	N.A.
SEPTIN9	rs11650934	Non-coding	N.A.	N.A.	0	No	N.A.
SNU13	rs6519270	Non-coding	N.A.	N.A.	N.A.	Yes	Fibroblasts, lung, whole blood, muscle, brain

CADD: Combined Annotation Dependent Depletion, REVEL: Rare Exome Variant Ensemble Learner, eQTL: expression quantitative trait loci.
